# A review of conjugation technologies for antibody drug conjugates

**DOI:** 10.1093/abt/tbaf010

**Published:** 2025-04-17

**Authors:** Qirui Fan, Hu Chen, Guoguang Wei, Ding Wei, Zekun Wang, Lin Zhang, Jun Wang, Marie Zhu

**Affiliations:** Department of Discovery & Development, WuXi XDC Co., Ltd, 520 Fute North Road, Pilot Free Trade Zone, Pudong New Area, Shanghai, 200131, China; Department of Discovery & Development, WuXi XDC Co., Ltd, 520 Fute North Road, Pilot Free Trade Zone, Pudong New Area, Shanghai, 200131, China; Department of Discovery & Development, WuXi XDC Co., Ltd, 520 Fute North Road, Pilot Free Trade Zone, Pudong New Area, Shanghai, 200131, China; Department of Discovery & Development, WuXi XDC Co., Ltd, 520 Fute North Road, Pilot Free Trade Zone, Pudong New Area, Shanghai, 200131, China; Department of Discovery & Development, WuXi XDC Co., Ltd, 520 Fute North Road, Pilot Free Trade Zone, Pudong New Area, Shanghai, 200131, China; Department of Discovery & Development, WuXi XDC Co., Ltd, 520 Fute North Road, Pilot Free Trade Zone, Pudong New Area, Shanghai, 200131, China; Department of Discovery & Development, WuXi XDC Co., Ltd, 520 Fute North Road, Pilot Free Trade Zone, Pudong New Area, Shanghai, 200131, China; Department of Discovery & Development, WuXi XDC Co., Ltd, 520 Fute North Road, Pilot Free Trade Zone, Pudong New Area, Shanghai, 200131, China

**Keywords:** antibody–drug conjugate (ADC), lysine conjugation, cysteine conjugation, enzymatic-tag conjugation, glycan remodeling conjugation, affinity peptide conjugation, non-canonical amino acids (ncAAs) conjugation

## Abstract

Antibody–drug conjugates (ADCs) have gained significant attention in biotherapeutics after several years of steady development. Among the multiple factors influencing ADC design, the conjugation method is one of the most critical parameters. This review classifies conjugation strategies into three categories: non-specific, site-specific but non-selective, and fully site-specific and selective methods. The characteristics; advantages and disadvantages; chemistry, manufacturing, and controls (CMC) potential; and clinical status of each conjugation strategy are discussed in detail. The site-specific and selective methods will yield more homogeneous ADC, which may influence the stability and pharmacokinetics (PK) profile of the ADC and then influence the final therapeutic outcome. Additionally, the review also explores challenges and future directions for developing novel conjugation strategies. This review presents the most prevalent conjugation techniques, providing a valuable resource for researchers in selecting conjugation technologies and advancing ADC development.

## Teaser

This review presents the most prevalent conjugation techniques used in clinical ADC pipelines or those with significant potential; compares the characteristics, advantages and shortcomings, CMC potential, and clinical status of each conjugation strategy; and discusses the potential challenges for further development of novel conjugation strategies.

## Introduction

Decades of advancements in antibody–drug conjugates (ADCs) and bioconjugates have revolutionized targeted therapies for various diseases. For example, ADCs—with cytotoxic molecules conjugated to antibodies—can effectively target by leveraging the antibodies’ specificity to tumor-associated proteins on the cell surface. Unlike traditional chemotherapy, ADCs selectively target tumor cells while sparing normal cells, making them highly desirable for cancer therapy. By the time this review is in preparation, there are 15 ADCs (conjugated with potent toxic payloads) on the market, approved by the US Food and Drug Administration (FDA), as well as the Chinese National Medical Products Administration and the Japanese Pharmaceuticals and Medical Devices Agency drug regulatory agencies (Table 1). With over 70 ADCs in clinical phase II/III trials, and more in phase I, further ADCs are expected to be commercialized, benefiting cancer patients in the years to come [1–4].

**Table 1:** Basic information on clinically approved ADCs conjugated with potent payloads

**Drug**	**Target**	**Company**	**Trade name**	**Payload**	**Connector**	**Conjugation strategies**	**Approval year**
Gemtuzumab ozogamicin	CD33	Pfizer/Wyeth	Mylotarg	N-acetyl-γ-calicheamicin (ozogamicin)	Hydrazone	Lysine conjugation	2000; 2017
Brentuximab vedotin	CD30	Seagen Genetics, Millennium/Takeda	Adcetris	MMAE^a^/auristatin	Maleimide	Interchain cysteine conjugation	2011
Trastuzumab emtansine	HER2	Genentech, Roche	Kadcyla	DM1^b^/maytansinoid	MCC^c^	Lysine conjugation	2013
Inotuzumab ozogamicin	CD22	Pfizer/Wyeth	Besponsa	N-acetyl- γ-calicheamicin (ozogamicin)	Hydrazone	Lysine conjugation	2017
Polatuzumab vedotin	CD79	Genentech, Roche	Polivy	MMAE/auristatin	Maleimide	Interchain cysteine conjugation	2019
Enfortumab vedotin	Nectin-4	Astellas/Seagen Genetics	Padcev	MMAE/auristatin	Maleimide	Interchain cysteine conjugation	2019
Trastuzumab deruxtecan	HER2	AstraZeneca/Daiichi Sankyo	Enhertu	Dxd^d^/camptothecin	Maleimide	Interchain cysteine conjugation	2019
Sacituzumab govitecan	Trop-2	Immunomedics	Trodelvy	SN-38/camptothecin	Maleimide	Interchain cysteine conjugation	2020
Belantamab mafodotin	BCMA	GlaxoSmithKline (GSK)	Blenrep	MMAF^e^/auristatin	Maleimide	Interchain cysteine conjugation	2020
Loncastuximab tesirine	CD19	ADC Therapeutics	Zynlonta	SG3199/PBD^f^ dimer	Maleimide	Interchain cysteine conjugation	2021
Tisotumab vedotin	Tissue factor	Seagen	Tivdak	MMAE/auristatin	Maleimide	Interchain cysteine conjugation	2021
Disitamab vedotin	HER2	RemeGen	Aidixi	MMAE	Maleimide	Interchain cysteine conjugation	2021
Mirvetuximab soravtansine	FRα	ImmunoGen	Elahere	DM4^g^	Sulfo-SPDB^h^	Lysine conjugation	2022
Sacituzumab tirumotecan	Trop2	Kelun Biotech/MSD	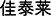 (Jiatailai)	Tirumotecan	2-(Methylsulfonyl)pyrimidine (KTHIOL™)	Interchain cysteine conjugation	2024
Datopotamab deruxtecan	Trop2	AstraZeneca/Daiichi Sankyo	Datroway	Dxd/camptothecin	Maleimide	Interchain cysteine conjugation	2024

^a^MMAE, monomethyl auristatin E.

^b^DM1, mertansine.

^c^MCC, 4-(*N*-maleimidomethyl)cyclo-hexane-1-carboxylate.

^d^Dxd, exatecan derivative.

^e^MMAF, monomethylauristatin F.

^f^PBD, pyrrolobenzodiazepine.

^g^DM4, maytansinoid DM4.

^h^Sulfo-SPDB, sulfo-sulfosuccinimidyl 4-(2-pyridyldithio) butyrate.

Multiple factors in ADC design affect its success, including antibody, linker, payload selection, site of drug attachment, and drug-to-antibody ratio (DAR), all of which play important roles. The method of drug attachment to an antibody is crucial; where and how the drug is linked affects the stability, efficacy, and pharmacokinetics (PK) of an ADC. This process is challenging because conjugation methods are limited by the vulnerability of bio-macromolecules involved. Additionally, reactions must be performed in an aqueous solution (with minimal organic solvent) and within a limited range of pH and temperature [5].

**Figure 1:**
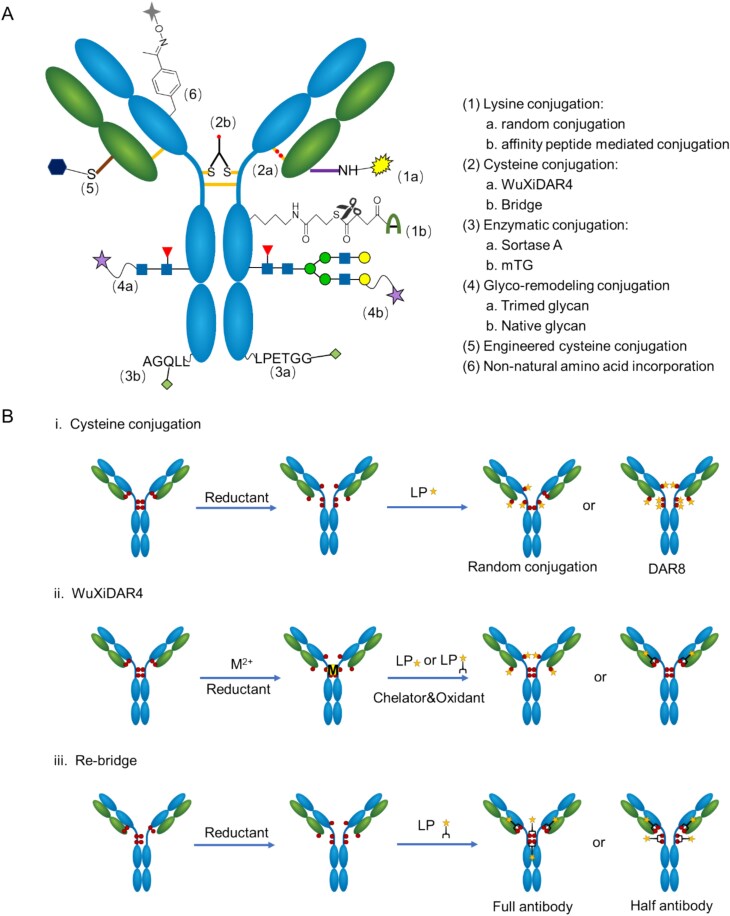
Scheme of site-specific conjugation techniques and interchain cysteine conjugation process. (A) Illustration of site-specific conjugation techniques, highlighting key reaction mechanisms and conjugation sites. (B) Illustration of interchain cysteine conjugation process with different strategies of (i) cysteine conjugation, (ii) WuXiDAR4, and (iii) re-bridge.

Conjugation methods are traditionally categorized into two groups: random conjugation and site-specific conjugation [6]. While this classification is straightforward and highlights the homogeneity improvement achieved by site-specific conjugation, it could imply a definitive superiority over random conjugation. Optimal conjugation locations vary for linker–payloads (LP) with different traits, meaning site-specific conjugation technologies might produce ADCs that are either superior or inferior to those made by random conjugation methods. In this review article, we divide these technologies into three categories. The first category is random conjugation (e.g. random lysine). The second category is site-specific but not site-selective, as the sites are confined by the conjugation technologies used. This category includes enzymatic-tag, glycan remodeling, affinity peptide conjugation, and “random” interchain cysteine conjugation (e.g. DAR4), as all the conjugation sites are limited to the four interchain cysteine pairs despite heterogeneous DAR distribution. The third category is site-specific and site-selective, modifying amino acid sequences such as engineered cysteine or ncAAs, providing various binding sites (Fig. 1A). Regarding CMC, random conjugation and interchain cysteine conjugation are generally simpler. Site-specific and site-selective conjugation technologies offer an improved homogeneity but may encounter more CMC challenges including protein expression issues (e.g. non-natural amino acids), additional purification steps required to remove impurities (e.g. enzymes), and additional analytical and quality control challenges (e.g. DAR distribution).

Due to scope limitations, this review does not provide a comprehensive review of all the available bioconjugation chemistries. Instead, it aims to present the most prevalent conjugation techniques used in clinical ADC pipelines or those with significant potential for a broader therapeutic window or more streamlined CMC processes. Our goal is to assist new researchers in selecting conjugation technologies that meet their requirements. For a more detailed review of bioconjugation technologies, please refer to the appended review references [7–9].

## Random conjugation

### Random lysine conjugation

Lysine conjugation is a well-established technique for producing ADCs, utilizing the approximately 40 solvent-accessible NH_2_ groups on lysine residues, which have high nucleophilicity in neutral solutions [10]. Electrophilic reagents primarily target these amino groups, allowing linker–payloads to attach without altering the antibody itself.

To date, five commercially available ADCs demonstrate the efficacy of lysine-based conjugation. Encouraged by this success, an extensive array of linkers has been developed to improve this process, including N-hydroxy succinimide (NHS) and its analogs [11], benzoyl fluoride [12], isothiocyanate [13], and squaramate ester [14].

Since these linkers are highly reactive with heteroatoms, they can also be quenched in water. In fact, the hydrolysis of NHS ester is one of the key factors that influences the LP equivalent in lysine conjugation. Additionally, less reactive linkers can react with -SH residues of cysteine under mild conditions, where the reaction with lysine is slow. For instance, sulfonyl can be used as a connector for cysteine [15], while sulfonyl acrylate can react with two cysteine residues to act as a bridge linker [16].

The high reactivity of lysine conjugation linkers initially led to their characterization as random modifications. However, the comparison of different batches of the same ADC done by Liu et al [17] found that the distribution of conjugation sites was found to be consistent under stable manufacturing conditions. Thus, the manufacturing process of current lysine-conjugated ADCs is reliable and repeatable, which is the cornerstone of their commercial availability.

However, varying conditions can produce diverse outcomes, such as highly heterogeneous DARs, which are linked to rapid clearance and toxicity. For example, Mylotarg®, with a heterogeneous DAR, was withdrawn from the market in 2010 due to its toxicity and safety concerns, including a high number of early deaths. It was re-approved in 2017 after redefining the patient population as well as a lower recommended dose and a different schedule. Meanwhile, the withdrawn of Mylotarg® has deeply influenced newer lysine-conjugated ADCs. Rather than using an unstable hydrazone linker and highly potent calicheamicin, linkers with higher robustness and less toxic maytansine analogs payloads were equipped. Changes in design enabled Kadcyla® and Elahere® to realize a higher dosage and a better therapeutic window than Mylotarg.

For less reactive linkers, under mild conjugation conditions, lysine conjugation can also yield homogeneous results, with the conjugation site being the most reactive lysine residue on the protein surface. For example, β-lactam or methylsulfone has been used to conjugate the K99 site in a hydrophobic pocket [18], while pentafluorophenol ester has been used to selectively conjugate the K188 site of kappa light chains using the K-lock™ technique [19]. It successfully empowered ADCs into clinical trials such as A166, ZV0203, and AMT-151 [20]. The phospha-Mannich reaction can selectively modify the K183 position of trastuzumab Fab [21]. Notably, -NH2 group reactivity on lysine is influenced by the local chemical environment on the protein surface, including factors such as counter-ions and solvation.

Overall, lysine conjugation remains a dependable method for ADC production, yielding chemically stable and reproducible products. Moreover, the development of site-specific conjugation techniques continues to enhance its potential for better homogeneity.

## Site-specific but not site-selective conjugation

### Interchain cysteine conjugation

Typically, an IgG1 antibody contains four pairs of interchain disulfide bonds in solvent-exposed areas. Eight free thiols can be obtained after reduction using reducing agents such as tris (2-carboxyethyl) phosphine (TCEP) and DL-dithiothreitol (DTT). Thiols, as nucleophiles, are softer and more amenable to Michael addition compared to SN2 reactions at lysine residues [22]. This property allows maleimide and its analogs to be used as connectors, enabling clean and nearly quantitative thiol-succinimide formation [23]. Such bioorthogonal chemistry is ideal for antibody modifications, producing variants with 0, 2, 4, 6, and 8 payloads [24]. Cysteine-mediated ADC heterogeneity is considerably less than that of lysine-based methods due to fewer thiol groups [25]. In cysteine conjugation technologies, the predominant approach involves random DAR4 conjugation. While other conjugation technologies including DAR8, WuXiDAR4, and disulfide re-bridging demonstrate site-specific conjugation capabilities. This review classifies all aforementioned techniques under interchain cysteine conjugation based on their mechanistic similarities in thiol group engagement. Compared to other conjugation methods, conjugates with maleimide stand out for simplicity, controllable conditions, and high yields (Fig. 1A). To date, maleimide conjugation with reduced interchain disulfides remains the predominant method for ADC construction. Ten out of the 15 commercialized ADCs with potent toxic payloads and most of the ADCs in the clinical stage have used maleimide conjugation technology (Table 1).

Maleimide-thiol conjugates are prone to reverse-Michael addition reactions, resulting in premature payload release through serum protein interactions. This reverse-Michael addition reaction can impact the stability of the ADCs in plasma and reduce their effectiveness and safety [26]. To address this issue, scientists have explored several strategies, including catalyzing the hydrolysis of maleimide by introducing additional groups such as N-aryl [27] and ortho amino [28] or replacing maleimide with ring-opening maleimide methyl ester [29] to avoid reverse-Michael addition reactions. Additionally, novel linkers such as KTHIOL™ [30], P5™ [31], and bromoacetamidecaproyl [32] have demonstrated enhanced thiol selectivity and resistance to retro-Michael addition reactions.

In addition, although the average DAR of the ADC products can be well-controlled by optimizing the amount of reduction reagent, the DAR distribution of ADC achieved through partial reduction and conjugation remains heterogeneous. Researchers from Daiichi Sankyo have found that conjugating at 4 °C increased the abundance of DAR4 species to about 55%, in which linker–payloads are primarily conjugated in the Fab region [33]. To improve the homogeneity of ADCs and to reduce the impact of unwanted species such as DAR0 and DAR8—both of which significantly affect ADC’s efficacy, PK, and toxicity—WuXi XDC has developed the WuXiDAR4™ conjugation platform to solve this problem.

WuXiDAR4™ uses a “hinge shielding” mechanism where metal ions such as Zn^2+^ are added during the reduction step, binding to the interchain disulfide bonds between the heavy chains of an IgG1 antibody. This selective binding ensures that only the disulfide bonds between the light and heavy chains are reduced, allowing linker–payloads to be conjugated at these positions, resulting in ADCs with improved homogeneity (over 70% DAR4 species). The homogeneity can be improved to over 95% with an additional column chromatography enrichment step. WuXiDAR4™ ADCs showed increased efficacy in cell-derived xenograft (CDX) models and higher tolerance in mice acute toxicity tests, suggesting a broader therapeutic window. Seven ADCs using this method have entered clinical phases. The process operates on native human IgG1 and is reliable and cost-effective, with a cost of goods comparable to conventional random conjugation [34]. (Fig. 1B).

Another method to obtain homogeneous products is by fully reducing four pairs of disulfide bonds and conjugating with linker–payloads, utilizing disulfide re-bridging technology. Disulfide re-bridging involves using dual thiol-reacting linkers to bind with a pair of cysteines simultaneously. Payloads can be straightly introduced through re-bridging disulfide bonds or connected via bioorthogonal reactions after introducing a highly reactive trigger. This method maintains the antibody's morphology and controls the reaction site, with the DAR of the products adjustable to approximately 4, 8, or 16 by varying the amounts of payloads on the linkers [35].

The most established reagents in disulfide re-bridging technology are disulfone compounds. In 2014, Godwin’s group from Abzena synthesized ADCs with a DAR4% of 78% using disulfone reagents, which demonstrated good serum stability and antitumor activity [36]. The OBI-999 developed by OBI Pharma Inc. employs similar principles and is currently in phase II clinical trials in Taiwan and the USA, having achieved the FDA orphan drug status for pancreatic cancer treatment [37]. Besides disulfone linkers, various other re-bridging linkers have been developed, such as new maleimide linkers [38], pyridazine diketone linkers [39], IDconnect™ [40], C-Lock™ [41], and CysLink [42]. Notably, 9MW2821 has entered phase 3 clinical trials with Mabwell’s IDconnect™ [40] and STI-6129 has advanced to phase 1/2 clinical trials with C-Lock™ developed by Sorrento Therapeutics [41].

However, the ADCs produced by re-bridging methods often result in a mixture of two isomers: “full-antibody” and “half-antibody” species (Section 3 of Fig. 2). The presence of “half-antibody” species may impact the antibody-dependent cell-mediated cytotoxicity (ADCC) activity of ADCs compared to their parent antibodies [43] Disulfide re-bridging technology continues to evolve, aiming to address the issue of “half-antibody” isomers, improve serum stability, and enhance the safety and effectiveness of these ADCs. Chudasama‘s group modified the structure of the linker–payload by combining the reducing agent TCEP with the pyridazinedione linker. This one-pot prepared ADC significantly reduced the formation of half-antibodies. However, the reagent demonstrated the same instability characteristics as TCEP, exhibiting poor stability and storage challenges [44]. This research team optimized the reaction sequence subsequently by first adding linker–payload followed by TCEP while lowering the conjugation temperature, which achieved improved yields of full-antibody species [45].

Regarding DAR8 technology, DS-8201 is the first and most well-known ADC approved by the FDA with 8 linker–payloads conjugated in interchain cysteines. However, whether fully reducing the interchain disulfide bonds affects the integrity and stability of antibodies remains controversial. Pfizer's research indicated that ADCs with high DAR experience negative effects on biophysical properties and thermal stability [46]. In contrast, Seagen's findings suggest that DAR8 ADCs maintain structure and stability despite full reduction and linking with PEG12-gluc-MMAE [47]. Additionally, Baker and Chudasama reported that fully reducing and capping the disulfides of trastuzumab significantly affected the CD16a kinetics, which is related to NK cell reactivity [33]. Thus, comprehensive structural evaluation is critical for assessing any negative impact on ADCs and their parental antibodies. In recent years, researchers focused on linker optimization to reduce hydrophobicity and aggregation propensity, thereby minimizing potential performance trade-offs in DAR8 ADCs. For example, ProfoundBio and Multitude Therapeutics have developed a series of exatecan derivatives with hydrophilic linkers. Their respective ADC candidates—Rinatabart Sesutecan and AMT253—have successfully overcome the challenge of exatecan's excessive hydrophobicity and potential aggregation risks on DAR8 ADCs [48, 49].

### Enzymatic-tag conjugation

Enzyme conjugation involves attaching payloads directly to antibodies by utilizing enzymes that recognize specific amino acid sequences. This technique offers high homogeneity in ADCs, showcasing its potential as an effective coupling method. Many enzymes require sequence engineering and structural adaptations of substrates such as antibodies. Clinically used enzymes include Sortase A (SrtA), a 30 kDa transpeptidase that cleaves LPXTG sequences to form a thioester acyl-enzyme intermediate, allowing peptide-LPXT to transfer to a substrate's N-terminus [50]. Notable examples include NBE-002 [51] (SMAC-Technology™), currently in phase 1/2 trials. Another example is the formylglycine-generating enzyme [52], which attaches to CXPXR sequences, converting cysteine to formylglycine, as seen with TRPH-222 [53] (SMARtag™), now in phase 1. Farnesyltransferase [54] modifies antibodies by adding isoprenoid groups to cysteine residues within a CaaX tag, with FS-1502 [55] (ConjuAll™) currently in phase 3. Preclinical stage enzymes include peptide asparaginyl ligases, tubulin–tyrosine ligase, trypsiligase, phosphopantetheinyl transferases, SpyLigase, and O6-Alkylguanine-DNA alkyltransferase (or SNAP-tag). Some enzymes, such as microbial transglutaminase (mTG) [56], target natural sites on antibodies, such as the Q295 site, catalyzing the formation of amide bonds between γ-carboxyamide of glutamine and a free amine group of a payload, with the representative molecule DP303 currently in phase III [57]. Successful development of enzyme conjugate molecules requires solving issues related to the PK of the conjugate products as well as the preparation aspects.

With the development of enzyme conjugation technology, research has found that the placement of modification tags on antibodies greatly influences both conjugation efficiency and overall product performance. Some research indicates that antibodies tagged on the heavy chain show superior titers and conjugation efficiency compared to those tagged on the light chain [58], while other studies suggest that light chain-tagged antibodies might offer effectiveness benefits [59, 60]. Currently, comprehensive data connecting ADC biophysical characteristics to functional outcomes are limited, and no standardized guidelines exist for selecting enzymes or optimal conjugation sites. Additionally, while antibody modifications can simplify conjugation processes, introducing heterologous peptides may increase immunogenicity risks [61]. This risk can be mitigated by using tags with low immunogenicity obtained through practice or calculation, or by employing tag-free enzyme conjugation technology [62]. (Selecting epitopes that are the natural structure of the antibody, or not requiring additional tags).

Researchers are advancing enzyme conjugation applications by enhancing the functions of genetically altered enzymes and refining the coupling processes to improve reaction efficiency, reduce reaction reversibility, and lower material costs. For example, wild-type SrtA has poor catalytic efficiency and reversible reactions, requiring a substantial excess of nucleophilic oligoglycine-modified substrates for effective ADC formation. To address this, researchers have modified SrtA to enhance its catalytic effectiveness [63, 64]. Additionally, transitioning from one-step to two-step coupling has improved the enzyme activity and minimized reaction reversibility. This method also holds promise for scaling up due to reduced costs and toxic waste generation, offering a novel approach to refining enzyme conjugation [65].

Enzyme coupling processes are often more complex than conventional techniques, requiring a broader range of materials and more complicated steps, which can significantly affect production cost and success likelihood. The selection of components—including enzymes, engineered antibodies, and small molecule payloads—must adhere strictly to Good Manufacturing Practice of Medical Products standards from the outset.

Furthermore, compared to traditional conjugation, enzyme conjugation introduces additional components such as conjugating enzymes, cofactors, and expression-related impurities, all of which have potential immunogenicity. Additional measures are necessary to eliminate catalytic enzymes and contaminants such as conjugating enzymes, cofactors, host cell DNA, and host cell protein from the final products. For example, additional multi-mechanism column purification methods and more complex and sensitive detection systems are required to control the residues of the aforementioned impurities. Additionally, experienced researchers are needed to evaluate the simplest production processes to reduce costs during the CMC stage.

Overall, enzyme conjugation is a powerful site-specific technology that has achieved numerous successes in the preclinical and clinical stages. Next-generation enzyme conjugation technologies can enhance product homogeneity through technological updates and optimized CMC processes. Through synergistic multi-dimensional strategies, we can systematically reduce immunogenicity while improving product homogeneity, ensuring the safety and efficacy of biologics such as ADCs. This enables dual breakthroughs in process stability and therapeutic safety.

### Glycan remodeling conjugation

Glyco-conjugation has garnered significant interest from both academia and industry in the recent decade. While most glycol-conjugation methods still rely on enzymes to facilitate the conjugation process, they do not require amino acid sequence engineering. Early techniques involved using sodium periodate (NaIO_4_) to oxidize the cis diols on glycans, creating aldehyde groups for subsequent modifications [66]. More recent strategies emphasize glycan remodeling, where native glycans (attached to the N297 site) are either modified or replaced with new ones, which can then be linked to functional linkers or linker–drug complexes. For example, Synaffix’s GlycoConnect™ technology uses an endoglycosidase to trim natural glycan isoforms, followed by the addition of an azido-modified galactose residue with a galactosyltransferase [67]. This modified glycan is then linked to a payload via click chemistry with a compatible handle. When used in conjugation with the HydraSpace linker [68], this method has seen widespread adoption in ADC development, as evidenced by six active clinical phase programs, including IBI-343 in phase 3 and several more in the preclinical stage [69, 70]. However, this process involves at least two enzymes and three conjugation steps, which can complicate development, reduce yield, and increase CMC costs. In the past few years, a promising glycan remodeling technology based on endo S2 (or its variants) emerged. This enzyme can deglycosylate and transfer glycosyl groups, enabling the attachment of a target molecule to the N297 site in one conjugation step. Huang’s group [71] and GeneQuantum’s team [72] pre-attach the glycan substrate to the linker–payload before ADC conjugation, using a single enzyme in one step. Wang’s team employs a dual-stage reaction process, initially incorporating an azido group enzymatically, followed by click chemistry to attach the linker–payload, which reduces enzyme use and reaction time [73]. Although not clinically validated yet, the endo S2 approach is appealing due to its reduced CMC cost. The various approaches to glycan remodeling are detailed in Table 2:

**Table 2:** Summary of various approaches to glycan remodeling

**Developer**	**Conjugation scheme**	**Clinical active ADC pipeline**
Synaffix [67]	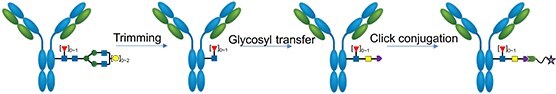	IBI-343, ADCT-601, ADCT-701, XMT-1660, MRG004, MGC026
Alphamab [74]	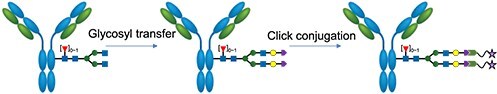	JSKN-003, JSKN-016
Daiichi Sankyo [75]	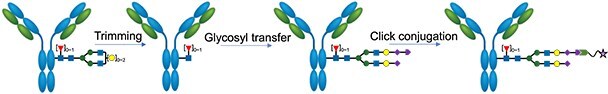	DS-9606a (tech licensed from GlycoT Therapeutics)
GlycoT Therapeutics/University of Maryland [73, 76]	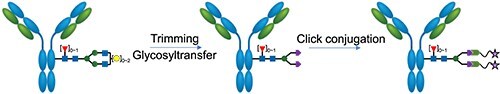	NA
GlycanLink [71]	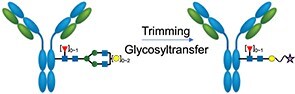	NA
GeneQuantum [72]	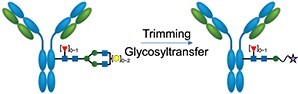	NA
GlycoTherapy [77]	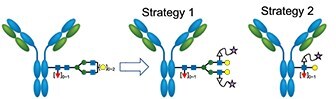	NA

Glycan remodeling technology has shown promising results in preclinical studies. For instance, Verkade et al. from Synaffix reported that their ADC (Brentuximab, DAR2-MMAE) achieved 7/7 complete responses in a Karpas-299 CDX model with a single dose of 1 mg/kg, while Adcetris (Brentuximab, DAR4-MMAE) was ineffective at the same dose [67]. The advantages of N297-glycan modification are evident; however, achieving optimal efficacy requires selecting the right combination of glycan type, linker, and DAR. The Huang group found that ADCs with trimmed glycans at DAR2 outperformed those with full glycans at DAR4 in CDX models. They theorize that compared to the ADCs with larger sizes of full glycans, the hydrophobic payloads in the trimmed glycan ADCs are better accommodated within the Fc cavity, thus showing better PK [78]. However, Wang’s studies did not show a clear preference for trimmed versus intact glycan forms in CDX models [76].

The impact of glycan on antibody Fc functions raises concerns. Generally, remodeling does not significantly affect the neonatal Fc receptor affinity, crucial for IgG PK. Huang’s group noted that Fcγ affinity is preserved in ADCs with intact glycans but not with trimmed ones [78]. While Fcγ is important for immunostimulatory ADCs, opinions differ for classical ADCs after internalization and lysosomal payload release. Some developers prefer maintaining IgG effector functions such as ADCC, complement-dependent cytotoxicity, and antibody-dependent cellular phagocytosis to enhance efficacy, while others believe these functions have minimal impact on tumor suppression and may lead to off-target toxicity, making intentional muting of effector functions a common approach.

Process development for glycan remodeling varies in complexity. Synaffix’s GlycoConnect uses two enzymes—endoglycosidase and galactosyltransferase—involving three reaction steps, which can complicate development, reduce yield, and increase CMC costs [67]. Alternatively, endo S2 (or its variants) combines deglycosylation and glycosyl transfer, simplifying the process.

### Affinity peptide conjugation

Affinity-directed conjugation uses a linker or linker–payload with an affinity peptide derived from the IgG binding site of protein A or G. This peptide selectively binds to specific sites on the Fc region, bringing it closer to certain lysine residues and enhancing the reaction speed between linker and -NH2 groups. To prevent side reactions with other reactive -NH2 groups, a mild linker should be used.

Typical conjugation sites are located near the K248/K288 or K337 residues of the Fc region [38]; while sequence modification of the affinity peptides can shift the conjugation site to the Fab region, this often results in reduced conjugation efficiency [79].

Some pioneering approaches have involved attaching linkers to the affinity peptide with stable covalent bonds, which then covalently link to an antibody to form ADCs [80]. However, large non-native peptides may interfere with Fc functionality by hindering FcRn binding [81], potentially reducing ADC internalization. Ishii-Watabe et al. noted that such peptide-bearing linkers might enhance ADCC, though PK was not assessed [82].

To minimize structural changes to the antibody, various traceless affinity-directed methods have been developed. Ajinomoto's AJICAP™ technology includes two generations: the first uses a disulfide bond that allows peptide cleavage by reductants [83], while the second uses a thioester that can be replaced directly by lysine during conjugation [84]. Abtis' AbClick™ shortens the affinity peptide sequence and utilizes a thioester for cleavage, capturing the freed thiol with N-tert-butyl maleimide for better efficiency and leaving a norbornene group for conjugation [85, 86]. Additionally, GlycanLink repositioned the connector from K8 to K10 to provide more space for direct conjugation of bulky payloads [87]. Alternative traceless methods have also been developed. Bode et al employed acyltrifluoroborate as a linker, which is removed after using a photocleavable linker on the affinity peptide [88]. Lee et al used an aldoxime to guide the linker to the K248 site [89].

## Site-specific and site-selective conjugation

### Engineered cysteine

Cysteine residues have been a cornerstone of protein and peptide modification for many years, due to the high reactivity of their thiol groups. The strategy of introducing drugs site-specifically where cysteines replace the original amino acids was pioneered by Junutula et al. [90], leading to the creation of cysteine-engineered antibodies known as ThioMabs.

Cysteine engineering is not universally applicable, as certain locations can lead to incorrect folding caused by unintended disulfide bond formation. Previous studies have shown that locations with minimal solvent exposure and a positive local charge—such as LC-V205C in trastuzumab—prevent maleimide exchange and create more stable ADCs through rapid maleimide hydrolysis [59]. Researchers have observed that the site of conjugation greatly influences the hydrophobic properties of the ADC; e.g. the HC-S239C conjugate exhibits very slow maleimide hydrolysis and enhanced stability [91]. Siddharth et al. [92] developed a mechanism-based PK/PD model for THIOMAB™ drug conjugates (TDCs), to help elucidate the impact of drug-loading, conjugation site, and subsequent deconjugation on PK and efficacy. Model results suggest that drug deconjugation rates, total antibody clearance, and tumor killing rates increase with DAR, and drug deconjugation occurs more readily from the heavy chain (HC-A114C) than the light chain (LC-V205C) sites used on these TDCs, consistent with *in vitro* and preclinical understanding. Ohri et al. [93] from Genentech conducted extensive research using high-throughput screening to evaluate the stability of ADCs produced from 648 different ThioMabs after substituting each position of an anti-HER2 antibody (trastuzumab) with cysteine. They identified 38 stable versions for both maleimide and disulfide conjugates (over 80% stable in ELISA) using MC-VC-PAB-MMAE and PDS-MMAE, respectively. Furthermore, they found that the *in vitro* plasma stability of site-specific ADCs correlates with *in vivo* stability. This phenomenon was also observed for ADCs formed using multiple different antibodies and different linker–payloads. An alternative strategy is to partially substitute cysteine in the hinge region with serine, thereby reducing reactive cysteine residues available for conjugation [94]. The sites of engineered cysteine used in clinic-stage ADCs are detailed in Table 3.

**Table 3:** Summary of engineered cysteine conjugation techniques in clinical stage

**Drug names**	**Engineered site**	**Highest phase**	**Drug targets**	**Linker**	**Payload**
**DMUC4064A**	HC-A114C	1	MUC16	Valine–citrulline	MMAE
**DCDS0780A**	HC-A114C	1	CD79b	Valine–citrulline	MMAE
**HDP-101**	HC-D265C	1/2	BCMA	Valine–alanine	Amanitin
**ABBV-321**	HC-S239C	1	EGFR	Valine–alanine	SGD-1882
**MEDI2228**	HC-S239C	1	BCMA	Valine–alanine	SG3199
**ABBV-176**	HC-S239C	1	Prolactin Receptor (PRLR)	Valine–alanine	SGD-1882
**BYON3521**	HC-P41C	1	c-MET	Valine–citrulline	DUBA
**BYON4413**	HC-P41C	1	CD123	Undisclosed	DUBA
**IMGN632**	HC-S442C	1/2	CD123	Alanine–alanine	DGN549
**ADCT-602**	HC-C220 LC-C214S/HC-C226S/HC-C229S	1/2	SIGLEC2	Valine–alanine	SG3199
**ADCT-401**	HC-C220 LC-C214S/HC-C226S/HC-C229S	1	PSMA	Valine–alanine	SG3199
**SC-003**	LC-C214	1	DPEP3	Valine–alanine	SG3199
**SC-002**	LC-C214	1	DLL3	Valine–alanine	SG3199
**SC-006**	LC-C214	1	RNF43	Valine–alanine	SC-DR003
**RG7861**	LC-V205C	1	*Staphylococcus aureus*	Valine–citrulline	dmDNA31
**ABBV-011**	LC-C214	1	SEZ6	Maleimide	Calicheamicin
**PYX-201**	HC-K290C/LC-K183C	1	Fibronectin extra-domain B	Valine–citrulline	PF-06380101
**JBH492**	HC-S152C/HC-S375C	1	CCR7	Disulfide	DM4

Site-specific ADCs typically present better preclinical profiles than non-site-specific ADCs; however, there have been limited data on their clinical advantages. Alex et al. [95] reported that the maximum administered dose for DCDS0780A (DAR2)—a TDC—is 4.8 mg/kg, which is at least double the dose of non-site-specific ADCs with the same linker–payload (MC-VC-PAB-MMAE) [96], such as polatuzumab vedotin (DAR 4). The effectiveness was comparable, with an objective response rate of 59% for TDCs and 56% for the non-site-specific ADCs. Nevertheless, ocular toxicities—which often led to therapy discontinuation—were more frequently observed in TDCs. Ocular adverse events (AEs) have been observed with payloads other than MMAE and are generally associated with ADCs featuring a stable linker. Joyce et al [97] reported similar observations in another TDC DMUC4064A, targeting MUC16. The recommended phase 2 dose for DMUC4064A (DAR 2) was identified as 5.2 mg/kg, compared to 2.4 mg/kg recommended for DMUC5754A (DAR 4), a non-site-specific ADC. An objective confirmed response was reported in 7 of 77 patients (9%) in the anti-MUC16 ADC study and in 16 of 65 patients (25%) in the anti-MUC16 TDC study. The most frequent AEs leading to a dose modification included blurred vision and peripheral neuropathy. The ocular toxicities observed in this study may be attributed to anti-tubulin effects and might be mediated by MUC16 expression in the eye’s epithelium, primarily managed with lubricating and moisturizing eye drops.

Engineered cysteines are typically prepared through reduction, re-oxidation, and conjugation steps. During re-oxidation, the majority of cysteines in the hinge region oxidize to form disulfide bonds, while the engineered ones remain available for conjugation. This process helps to avoid potential issues such as misshapen monoclonal antibodies or fragments.

Ruud and colleagues [98] at Byondis B.V. have introduced 2-(diphenylphosphino) benzenesulfonic acid, a selective reducer that targets engineered cysteines while preserving interchain disulfides. Renée from Pfizer [99] reported an innovative manufacturing approach that utilizes cysteine metabolic engineering in Chinese hamster ovary cells and novel cysteine capping techniques. This approach results in superior-quality ADCs characterized by homogeneous DAR, intact native hinge disulfides, and fewer fragments. These features may have beneficial effects on therapeutic efficiency.

Additionally, efforts are being made to identify optimal sites for producing uncapped monoclonal antibodies that maintain free cysteines throughout cell culture and purification processes. Examples of such sites include LC-Q124C [100] and LC-Q166C [99].

### Non-canonical amino acids

In addition to conjugating with native residues, antibodies can be site-specifically modified with the help of bioorthogonal reactions such as ketone condensation and strain-promoted azide-alkyne cycloaddition (SPAAC) etc. T Genetic code expansion (GCE) technology has enabled the co-translational incorporation of ncAA with bioorthogonal residues into antibodies at specific sites [101]. This is achieved using a host-orthogonal aminoacyl-tRNA synthetase/tRNA pair to incorporate the related ncAA into the target protein at a pre-mutated nonsense codon, typically a TAG stop codon [102].

Building on the groundbreaking and pioneering work, Ambrx has successfully prepared ketone-bearing antibodies using ncAA pAcF with their proprietary EuCODE expression platform [103]. Anti-Her2 and anti-PSMA antibodies were prepared in this approach. Corresponding ADCs were made separately by conjugating them with a hydroxylamine-capped linker–payload under acidic conditions, yielding ADCs with a DAR of approximately 2. These ADCs have shown promising results in both pre-clinical studies and clinical trials [104, 105]. Furthermore, research has found that these ADCs—generated through ketone condensation—exhibit improved PK, pharmacodynamics, and safety profiles compared to analogous heterogeneous ADCs. The easily performed conjugation process of these ADCs is undoubtedly advantageous, and the likelihood of successful development of these ADCs seems higher than others. However, it is important to recognize that ADCs of this type have been relatively understudied, and greater efforts from Veraxa and Brickbio—recently established ADC biotech companies—should be devoted to research regarding this technology [106, 107].

In spite of the promising pharmaceutical prospects, the low expression of antibodies based on GCE technology is a major drawback. Full-length ncAA-modified antibodies require efficient stop codon suppression to compete effectively against endogenous release factors [108–110]. Due to the intrinsic transcription termination mechanism and the potential inefficiency of the exogenous ncAA pairs, the read-through efficiency of the stop codon is typically much lower than the theoretical 100% read-through efficiency of natural amino acids.

To address this issue, scientists from MedImmune conducted extensive optimizations on the expression systems. Detailed optimizations were performed in both cell line construction and cell culture process development procedures. Under the best expression conditions, titers of >100 mg/L for transient systems and 1.5 ~ 2.5 g/L for stable expressions were achieved. Utilizing their sophisticated expression techniques, both azide- and diene-bearing mAbs were successfully expressed, enabling efficient ADC preparation by SPAAC or Diels–Alder reaction with improved kinetics under neutral pH.

Sutro Biopharma offered another potential solution for the efficient expression of ncAA-bearing mAbs through their sophisticated cell-free expression platform, XpressCF+ [111]. Antibodies modified by up to eight ncAAs—although in aglycosylated forms—were expressed up to g/L within hours [112]. Although the ncAA read-through efficiency still existed, the application of the prokaryotic cell-free system greatly reduced the overall expression cost. These ADCs with different payloads were prepared and tested [113], and they have shown superior therapeutic outcomes, warranting further evaluation in various-stage clinical trials.

## Concluding remarks

Identifying optimal conjugation sites and chemistries for specific antibody/linker–payload combinations remains a significant challenge due to the broad implications of conjugation technologies on ADC properties. These implications include binding, internalization, payload release, PK, effector functions, and others. Although newer conjugation technologies often demonstrate better efficacy and safety profiles, there is frequently a gap in translatability between preclinical and clinical studies. For instance, ADCs with greater stability often demonstrate better efficacy in xerograph models. Additionally, cynomolgus monkey toxicity studies may not reveal potential on-target toxicity. To conclude all these remarkable properties, we summarize a pros and cons table (Table 4) to compare the advantages and weaknesses of the various conjugation technologies.

**Table 4:** Summary of pros and cons of conjugation techniques

**Conjugation techniques**		**Pros**	**Cons**
Lysine conjugation	Random conjugation	① Efficient and simple reaction and purification process. ② Good product stability	Poor homogeneity [10]
	Affinity peptide-mediated conjugation	① Same product stability as random conjugation [80] ② Site-specific conjugation [80]	Equivalent or more affinity peptide needed [89]
Cysteine conjugation	Random conjugation	① Simple reaction with high yield ② Low cost	Poor homogeneity [24]
	Site-specific conjugation	① Simple reaction with high yield ② High homogeneity	WuXiDAR4: only available for IgG1 [34]
			Re-bridge: half-antibody and mismatch [43]
			DAR 8: lower compatibility with hydrophobic LP [46]
Enzymatic conjugation		① High homogeneity [51] ② Available in versatile antibodies [55]	① Extra immunogenicity risks [61] ② Additional purification and quality control processes requirement [61]
Glyco-remodeling conjugation		Favorable PK and efficacy [114]	① Costly CMC [67] ② Lack of effector functions (may not always be a con)
Engineered cysteine conjugation		① High homogeneity [90] ② Share the same LP with random cysteine conjugation	① Complex process [98] ② Limited number of conjugation sites [115]
Non-canonical amino acid incorporation		Homogeneity in the manufacturing process	Low antibody expression titer, which causes huge efforts in cell line development and cell culture process development [116]

Moreover, novel conjugation technologies can present unexpected challenges in the CMC process, even if the overall process appears more straightforward. As a result, some developers prefer to use clinically validated conjugation technologies, particularly when their pipelines already carry risks associated with novel targets and/or payload mechanisms of action.

Despite recent advances in ADC development, numerous questions in this field remain unresolved. As our understanding of ADC complexities deepens and we accumulate clinical data from more ADCs employing advanced conjugation methods, it is anticipated that new and more suitable conjugation techniques will emerge. These advancements will address unmet clinical needs.

## Data Availability

No new data were generated or analyzed in this review.
